# Evaluation of toll-like receptors as prognostic biomarkers in gastric cancer: high tissue TLR5 predicts a better outcome

**DOI:** 10.1038/s41598-019-49111-2

**Published:** 2019-08-29

**Authors:** Aaro Kasurinen, Jaana Hagström, Alli Laitinen, Arto Kokkola, Camilla Böckelman, Caj Haglund

**Affiliations:** 1 Translational Cancer Medicine Research Programme, University of Helsinki, Helsinki, Finland; 2 Department of Pathology and Oral Pathology, University of Helsinki and Helsinki University Hospital, Helsinki, Finland; 3 Department of Surgery, University of Helsinki and Helsinki University Hospital, Helsinki, Finland

**Keywords:** Prognostic markers, Gastric cancer

## Abstract

Toll-like receptors (TLRs), key proteins in innate immunity, appear to contribute to the inflammatory environment in carcinogenesis. Thus, we aimed to evaluate the tissue expressions of TLR1, TLR2, TLR4, TLR5, TLR7, and TLR9 as potential prognostic biomarkers in gastric cancer. We applied immunohistochemistry to study tissue samples from 313 patients operated on for gastric adenocarcinoma between 2000 and 2009 at the Department of Surgery, Helsinki University Hospital, Finland. A high expression of each TLR studied associated with the high expression of each other and with the intestinal-type histology (p < 0.001 for all). Five-year disease-specific survival among patients with a high TLR5 was 53.4% (95% confidence interval [CI] 43.4–63.4), whereas among patients with a low TLR5 it was 37.6% (95% CI 30.0–45.2; p = 0.014). A high TLR5 expression functioned as a marker of a better prognosis, particularly among those with a stage II disease (hazard ratio [HR] 0.33; 0.13–0.83; p = 0.019) or an intestinal-type cancer (HR 0.58; 95% CI 0.34–0.98; p = 0.043). In this study we show, for the first time, that a high TLR5 tissue expression may identify gastric cancer patients with a better prognosis, particularly among those with a stage II disease or an intestinal-type cancer.

## Introduction

Gastrointestinal tract malignancies cause more cancer-related deaths worldwide than any other form of cancer, accounting for more than 20% of all cancer-related fatalities^1^. Late diagnosis, often occurring already at an advanced disease stage, and a high recurrence risk result in poor prognoses.

Chronic inflammation contributes to carcinogenesis in the gastrointestinal tract, and several biomarkers have been studied in attempts to further clarify the pathology behind inflammation-associated cancers, including the toll-like receptors (TLRs)^2–4^. TLRs, a family of transmembranous pattern recognition receptors, play a crucial role in innate immunity. These receptors are expressed on antigen-presenting cells in the first line of defence, such as on the macrophages and dendritic cells, and activated by pathogen-associated molecular patterns^5^. In cancer, TLRs contribute to the inflammatory environment via activation by damage-associated molecular patterns^6^. However, TLRs may assume a heterogeneous role in cancer biology, since they appear to both induce antitumour factors and in different contexts promote procancerous mechanisms^7^. A sequence of increasing TLR2, TLR4, and TLR5 expression levels was observed with progression from normal gastric mucosa to pre-cancerous lesions, gastric dysplasia, and ultimately to gastric adenocarcinoma^8^. The highest TLR expression levels were found in dysplastic lesions, suggesting that TLRs may play a specific role in gastric cancer development.

TLR4 represents the most widely studied TLR in gastric cancer, and its polymorphism may associate with an increased risk of gastric cancer^9,10^. Furthermore, TLR4 signalling activation in gastric cancer cells by lipopolysaccharides increase the risk of metastasis^11^. In a study among 106 gastric adenocarcinoma patients, TLR3, TLR4, and TLR9 were highly expressed in gastric cancer tissues and survival worsened among patients with a high TLR3 expression^12^. Moreover, TLR2 expression in gastric cancer has been linked to metastatic disease and increased invasion^13^. In addition, TLR5 activation by flagellin, the major structural protein in bacterial flagellum, increases the proliferation of gastric cancer cells^14^. Subsequent TLR5 antagonism appeared to cancel the effect, suggesting that TLR5 signalling clearly contributes to the proliferation of gastric cancer cells. Yet, administering imiquimod (a TLR7 agonist) to gastric cancer cells results in a reduced proliferation^15^. In that study, TLR7 expression was low in gastric cancer cells compared to levels in adjacent healthy tissue. Genetic variations of TLR1 combined with an *Helicobacter Pylori* (*H*. *pylori*) infection predispose an individual to develop gastric cancer^16^. However, TLR1 remains unstudied using immunohistochemistry. TLRs are promising biomarkers, yet due to their diverse functions, further research is needed to clarify their roles in gastric cancer.

Therefore, in this study, we aimed to explore the tissue expression of TLR1, TLR2, TLR4, TLR5, TLR7, and TLR9 as potential prognostic biomarkers in gastric cancer patients, and to examine their associations with several clinicopathological variables.

## Methods

### Patients

We retrospectively studied 313 patients operated on for gastric cancer between 2000 and 2009 in the Department of Surgery, Helsinki University Hospital. Each gastric tumour was histologically confirmed as gastric adenocarcinoma by a pathologist of the Helsinki University Hospital. Individuals undergoing surgery were consecutively included in our patient cohort; we excluded patients with a history of malignant disease or any synchronous cancers. The median age at the time of surgery was 67.4 years (interquartile range [IQR] 57.1–76.5) and 152 (48.6%) were male. The median follow-up time was 2.3 years, with 66 (21.1%) patients alive at the end of follow-up. Living data until September 2017 were obtained from patient records, the Population Register Centre of Finland, and Statistics Finland. The five-year disease-specific survival for all patients was 43.3% (95% confidence interval [CI] 37.4–49.2). We used the seventh version of the TNM classification for disease staging^17^.

The Surgical Ethics Committee of Helsinki University Hospital approved our study (Dnro HUS 226/E6/ 06, extension TMK02 §66 17 April 2013). Permission to study archived tissue samples without individual consent was granted by the National Supervisory Authority of Welfare and Health (Valvira Dnro 10041/06.01.03.01/2012).

### Tissue samples and immunohistochemistry

We applied the same immunohistochemical staining protocol to each TLR. A tissue microarrayer (TMA Grand Master, 3D Histech Ltd, Budapest, Hungary) was used to punch four 1.0-mm cores from each sample, embedding them in a recipient block of paraffin. The TMA samples were subsequently cut in 4-µm sections for the immunohistochemical staining, resulting in four 1.0-mm tissue microarray spots per patient. The slides were deparaffined, pre-warmed in a PT module (LabVision UK Ltd, UK) to 65 °C, and treated for 20 min in 98 °C for antigen retrieval (Tris-EDTA buffer; pH 9.0 or Tris-Hcl buffer; pH 8.5). The staining was performed in an Autostainer 480 (LabVision) with the Dako detection system (Dako REAL EnVision Detection System, Peroxidase/DAB+, Rabbit/Mouse [Dako, Glostrup, Denmark]). Endogenous peroxidases were blocked with the 0.3% Dako REAL Peroxidase-Blocking Solution. The slides were then incubated with the primary antibody, using the following primary antibodies: TLR1 rabbit polyclonal 200 µg/ml (1:100, 1 hr, sc-30000, Santa Cruz Biotechnology, Dallas, TX, USA), TLR2 rabbit polyclonal 200 µg/ml (1:200, over-night (O/N), sc-10739, Santa Cruz Biotechnology), TLR4 mouse monoclonal IgG1 200 µg/ml (1:2000, 1 hr, sc-293072, Santa Cruz Biotechnology), TLR5 mouse monoclonal 0.1 mg/ml (1:100, 1 hr, NBP2-24787, Novus Biologicals, Centennial, CO, USA), TLR7 rabbit polyclonal 1.0 µg/ml (1:500, 1 hr, NBP2-24906, Novus Biologicals), and TLR9 mouse monoclonal 100 µg/ml (1:300, O/N, sc-52966, Santa Cruz Biotechnology). Finally, the samples were incubated with the peroxidase-conjugated Dako REAL EnVision/HRP, Rabbit/Mouse (ENV) secondary antibody for 30 min, visualised using the Dako REAL DAB+ Chromogen (10 min), and counterstained with Meyer’s hematoxylin.

### Scoring of immunoreactivities

**Figure 1:**
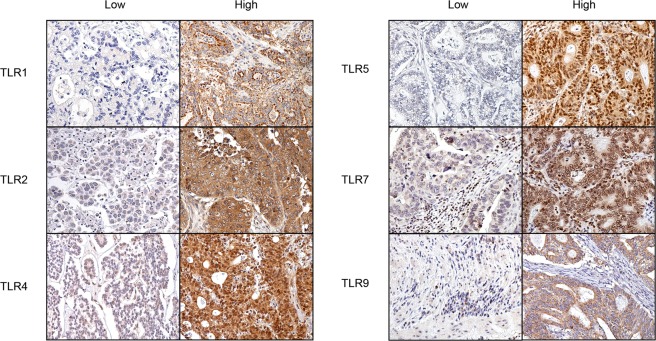
Representative images of immunohistochemistry demonstrating gastric cancer tumours with low versus high TLR immunoreactivities. Original magnification was 20×.

We scored TLR1 immunoreactivity on 282 tissue cores, TLR2 on 275 cores, TLR4 on 281 cores, TLR5 on 277 cores, TLR7 on 268 cores, and TLR9 on 277 cores. We excluded cores lacking cancer tissue. Among all TLRs, reactivity was observed both in the nuclei and in the cytoplasm. We quantified TLR expression by scoring the cancer cells’ cytoplasmic staining intensity. A score of 3 indicated strong staining, 2 moderate, 1 weak, and 0 signified the absence of staining. A total of four tissue cores per tumour sample were evaluated, from which we selected the tumour core with the highest score to represent each patient in the statistical analyses. For the final analyses, data were divided into two categories: high expression (strong or moderate immunoreactivity) and low expression (weak or no immunoreactivity) groups (Fig. 1). All tissue microarray cores were scored by two independent researchers, including an experienced pathologist from the Department of Pathology and Oral Pathology at the University of Helsinki (Aa.K. and J.H.), both blinded to the clinical data. Tissue core scores with any difference between researchers were re-evaluated, and the final score was reached through discussion and consensus.

### Statistical analyses

Associations and correlations were evaluated using the Pearson’s chi-squared test and the Spearman’s rank correlation test. We created the survival curves using the Kaplan–Meier method and compared them using the log-rank test. Disease-specific survival was determined from the date of surgery until death from gastric cancer or until the end of the follow-up period. We applied the Cox proportional hazard model to calculate the hazard ratios for the uni- and multivariate survival analyses. For the multivariate survival analysis, we included age, stage, the Laurén classification, and TLR5 expression in our model. Stage was processed as a categorical covariate, and we found no significant interaction terms. For all analyses, we considered a two tailed p < 0.05 as statistically significant, and for all statistical analyses we used IBM SPSS Statistics version 24.0 for Mac (IBM Corporation, Armonk, NY, USA).

## Results

### Associations between TLR expression and clinicopathological variables

Table 1 summarises the distribution of immunoreactivities. A high TLR2 and a high TLR4 expression associated with pT2–4 tumours (p = 0.043; p = 0.049; Table 2), and a high TLR7 expression associated with pT3 tumours (p = 0.025; Table 3). In addition, a high TLR7 expression associated with stage II disease (p = 0.030) and with being male (p = 0.021). A high TLR5 and a high TLR9 expression associated with an older age (p = 0.028; p = 0.001). Furthermore, a high expression of each TLR studied associated with the intestinal-type cancer (p < 0.001 for all). Lastly, a high expression of each TLR studied associated with high expressions of all other TLRs, whilst comparisons also revealed weak to moderate positive correlations (p < 0.001 for all; Table 4 and Supplementary Table 1).

**Table 1:** Distribution of immunoreactivity for TLR1, TLR2, TLR4, TLR5, TLR7, and TLR9.

	Immunoreactivity				
	Strong (%)	Moderate (%)	Weak (%)	Absent (%)	Total
TLR1	29 (10.3)	115 (40.8)	120 (42.6)	18 (6.4)	282
TLR2	44 (16.0)	122 (44.4)	90 (32.7)	19 (6.9)	275
TLR4	74 (26.3)	106 (37.7)	77 (27.4)	24 (8.5)	281
TLR5	11 (4.0)	98 (35.4)	114 (41.2)	54 (19.5)	277
TLR7	30 (11.2)	95 (35.4)	108 (40.3)	35 (13.1)	268
TLR9	13 (4.7)	130 (46.9)	115 (41.5)	19 (6.9)	277

Abbreviations: TLR = Toll-like receptor.

**Table 2:** Association of TLR1, TLR2, and TLR4 expressions with clinicopathologic variables in 313 gastric cancer patients.

	TLR1			TLR2			TLR4		
	Low (%)	High (%)	p value^a^	Low (%)	High (%)	p value^a^	Low (%)	High (%)	p value^a^
Age, years									
<67	76 (54.3)	64 (45.7)	0.074	61 (44.9)	75 (55.1)	0.080	55 (39.3)	85 (60.7)	0.245
≥67	62 (43.7)	80 (56.3)		48 (34.5)	91 (65.5)		46 (32.6)	95 (67.4)	
Gender									
Male	65 (47.4)	72 (52.6)	0.626	50 (36.8)	86 (63.2)	0.336	51 (37.2)	86 (62.8)	0.662
Female	73 (50.3)	72 (49.7)		59 (42.4)	80 (57.6)		50 (34.7)	94 (65.3)	
Stage									
I	22 (40.7)	32 (59.3)	0.230	24 (48.0)	26 (52.0)	0.576	24 (44.4)	30 (55.6)	0.119
II	30 (46.2)	35 (53.8)		23 (35.4)	42 (64.6)		17 (26.2)	48 (73.8)	
III	59 (56.7)	45 (43.3)		40 (39.2)	62 (60.8)		35 (34.0)	68 (66.0)	
IV	27 (46.6)	31 (53.4)		22 (38.6)	35 (61.4)		25 (43.1)	33 (56.9)	
Tumour classification (pT)									
pT1	19 (45.2)	23 (54.8)	0.912	23 (57.5)	17 (42.5)	0.043	21 (50.0)	21 (50.0)	0.049
pT2	20 (47.6)	22 (52.4)		11 (27.5)	29 (72.5)		13 (31.0)	29 (69.0)	
pT3	42 (48.3)	45 (51.7)		31 (36.5)	54 (63.5)		23 (26.7)	63 (73.3)	
pT4	57 (51.4)	54 (48.6)		44 (40.0)	66 (60.0)		44 (39.6)	67 (60.4)	
Lymph node metastasis (pN)									
pN0	40 (43.5)	52 (56.5)	0.251	38 (43.2)	50 (56.8)	0.415	33 (35.9)	59 (64.1)	0.745
pN1–3	92 (50.8)	89 (49.2)		68 (38.0)	111 (62.0)		61 (33.9)	119 (66.1)	
Distant metastasis (pM)									
pM0	111 (49.6)	113 (50.4)	0.648	87 (39.9)	131 (60.1)	0.857	76 (34.1)	147 (65.9)	0.202
pM1	27 (46.6)	31 (53.4)		22 (38.6)	35 (61.4)		25 (43.1)	33 (56.9)	
Laurén classification									
Intestinal	37 (32.7)	76 (67.3)	<0.001	21 (18.6)	92 (81.4)	<0.001	22 (19.5)	91 (80.5)	<0.001
Diffuse	101 (59.8)	68 (40.2)		88 (54.3)	74 (45.7)		79 (47.0)	89 (53.0)	

Abbreviations: TLR = Toll-like receptor.

^a^Pearson’s Chi–squared test.

**Table 3:** Association of TLR5, TLR7, and TLR9 expressions with clinicopathologic variables in 313 gastric cancer patients.

	TLR5			TLR7			TLR9		
	Low (%)	High (%)	p value^a^	Low (%)	High (%)	p value^a^	Low (%)	High (%)	p value^a^
Age, years									
<67	92 (67.2)	45 (32.8)	0.028	74 (56.5)	57 (43.5)	0.315	80 (58.0)	58 (42.0)	0.001
≥67	76 (54.3)	64 (45.7)		69 (50.4)	68 (49.6)		54 (38.8)	85 (61.2)	
Gender									
Male	75 (56.0)	59 (44.0)	0.123	61 (46.2)	71 (53.8)	0.021	60 (44.4)	75 (55.6)	0.202
Female	93 (65.0)	50 (35.0)		82 (60.3)	54 (39.7)		74 (52.1)	68 (47.9)	
Stage									
I	29 (54.7)	24 (45.3)	0.255	35 (71.4)	14 (28.6)	0.030	24 (46.2)	28 (53.8)	0.961
II	36 (55.4)	29 (44.6)		28 (43.8)	36 (56.3)		31 (47.7)	34 (52.3)	
III	64 (62.1)	39 (37.9)		53 (52.5)	48 (47.5)		52 (50.5)	51 (49.5)	
IV	39 (70.9)	16 (29.1)		27 (50.9)	26 (49.1)		27 (48.2)	29 (51.8)	
Tumour classification (pT)									
pT1	22 (52.4)	20 (47.6)	0.616	28 (73.7)	10 (26.3)	0.025	20 (48.8)	21 (51.2)	0.260
pT2	24 (60.0)	16 (40.0)		20 (51.3)	19 (48.7)		14 (34.1)	27 (65.9)	
pT3	52 (60.5)	34 (39.5)		37 (44.0)	47 (56.0)		43 (50.6)	42 (49.4)	
pT4	70 (64.2)	39 (35.8)		58 (54.2)	49 (45.8)		57 (51.8)	53 (48.2)	
Lymph node metastasis (pN)									
pN0	48 (53.3)	42 (46.7)	0.090	49 (56.3)	38 (43.7)	0.483	45 (50.0)	45 (50.0)	0.634
pN1–3	114 (64.0)	64 (36.0)		90 (51.7)	84 (48.3)		84 (46.9)	95 (53.1)	
Distant metastasis (pM)									
pM0	129 (58.1)	93 (41.9)	0.082	116 (54.0)	99 (46.0)	0.694	107 (48.4)	114 (51.6)	0.978
pM1	39 (70.9)	16 (29.1)		27 (50.9)	26 (49.1)		27 (48.2)	29 (51.8)	
Laurén classification									
Intestinal	49 (43.4)	64 (56.6)	<0.001	33 (29.7)	78 (70.3)	<0.001	26 (23.2)	86 (76.8)	<0.001
Diffuse	119 (72.6)	45 (27.4)		110 (70.1)	47 (29.9)		108 (65.5)	57 (34.5)	

Abbreviations: TLR = Toll-like receptor.

^a^Pearson’s Chi–squared test.

**Table 4:** Correlation of TLR1, TLR2, TLR4, TLR5, TLR7, and TLR9 expressions among each other in 313 gastric cancer patients.

	TLR1		TLR2		TLR4		TLR5		TLR7	
	r_s_	p value	r_s_	p value	r_s_	p value	r_s_	p value	r_s_	p value
TLR2	0.314	<0.001								
TLR4	0.293	<0.001	0.436	<0.001						
TLR5	0.345	<0.001	0.272	<0.001	0.212	<0.001				
TLR7	0.296	<0.001	0.401	<0.001	0.332	<0.001	0.253	<0.001		
TLR9	0.422	<0.001	0.384	<0.001	0.361	<0.001	0.267	<0.001	0.338	<0.001

Abbreviations: TLR = Toll-like receptor, r_s_ = Spearman’s rank correlation coefficient.

### Survival analyses

**Figure 2:**
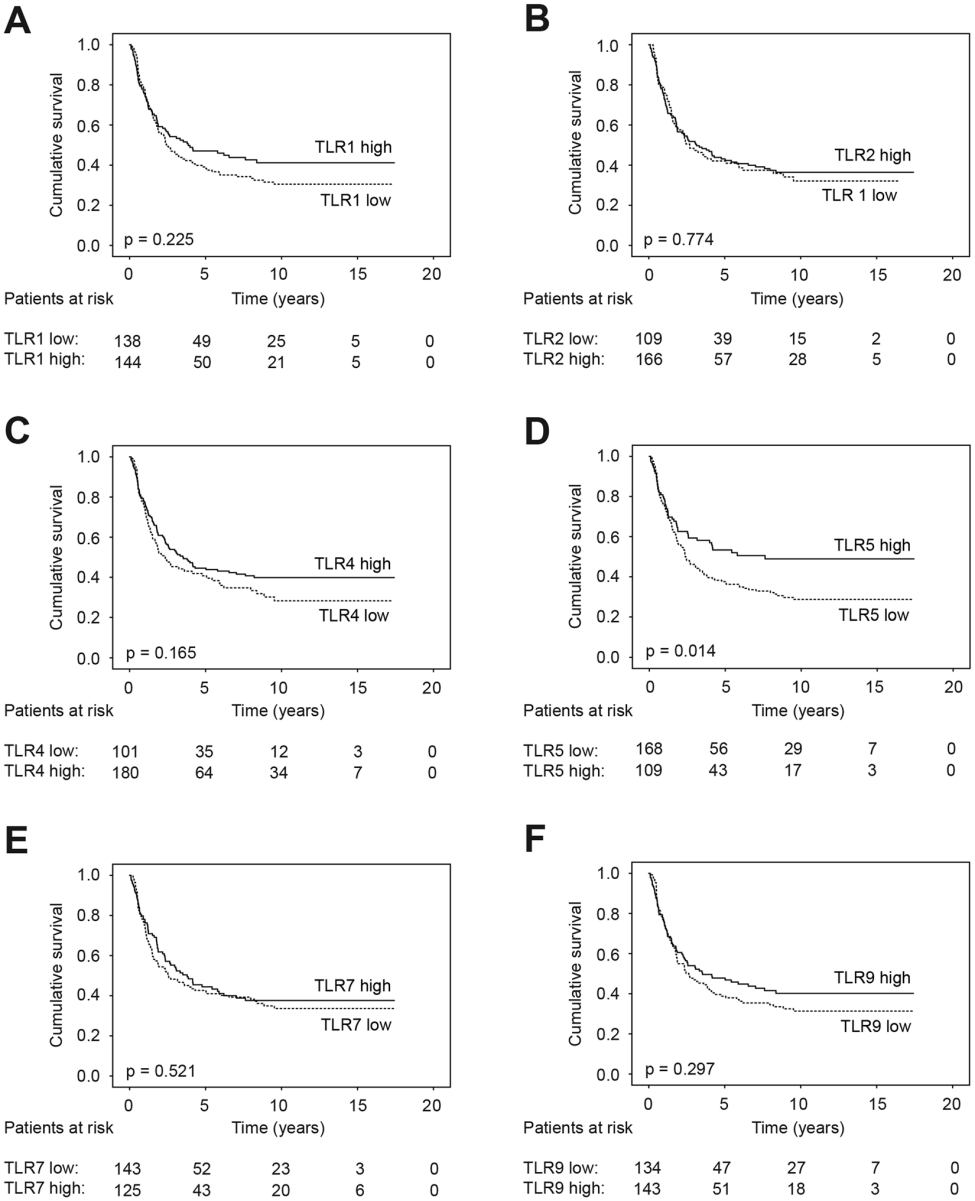
Gastric cancer patients’ disease-specific survival according to the Kaplan–Meier method, with p values for significance based on the log-rank test. High (**A**) TLR1, (**B**) TLR2, (**C**) TLR4, (**D**) TLR5, (**E**) TLR7, and (**F**) TLR9 expressions compared to low expressions.

The five-year disease-specific survival amongst gastric cancer patients with a high TLR5 expression was 53.4% (95% CI 43.4–63.4), compared to 37.6% (95% CI 30.0–45.2) among those with a low TLR5 expression (p = 0.014; Table 5 and Fig. 2D). TLR1, TLR2, TLR4, TLR7, and TLR9 expressions did not function as significant prognostic biomarkers across the entire cohort (Fig. 2). In the multivariate survival analysis, significant prognostic factors consisted of age, stage, and the Laurén classification (Table 5).

**Table 5:** Uni- and multivariate survival analyses for 313 gastric cancer patients.

	Univariate			Multivariate		
	HR	95% CI	p value	HR	95% CI	p value
Age, years						
<67	1.00			1.00		
≥67	1.33	1.00–1.79	0.054	2.60	1.85–3.66	<0.001
Stage						
I	1.00			1.00		
II	5.44	2.25–13.1	<0.001	4.86	1.85–12.8	0.001
III	15.7	6.85–36.1	<0.001	17.3	6.99–43.0	<0.001
IV	46.2	19.6–109	<0.001	63.2	24.5–163	<0.001
Laurén classification						
Intestinal	1.00			1.00		
Diffuse	1.45	1.06–1.98	0.020	1.53	1.09–2.17	0.016
TLR1						
Low	1.00					
High	0.83	0.61–1.13	0.225			
TLR2						
Low	1.00					
High	0.96	0.70–1.31	0.774			
TLR4						
Low	1.00					
High	0.80	0.59–1.10	0.166			
TLR5						
Low	1.00			1.00		
High	0.66	0.47–0.92	0.014	0.73	0.52–1.05	0.086
TLR7						
Low	1.00					
High	0.90	0.66–1.24	0.521			
TLR9						
Low	1.00					
High	0.85	0.62–1.16	0.298			

Abbreviations: TLR = Toll-like receptor, CI = Confidence interval, HR = Hazard ratio.

**Figure 3:**
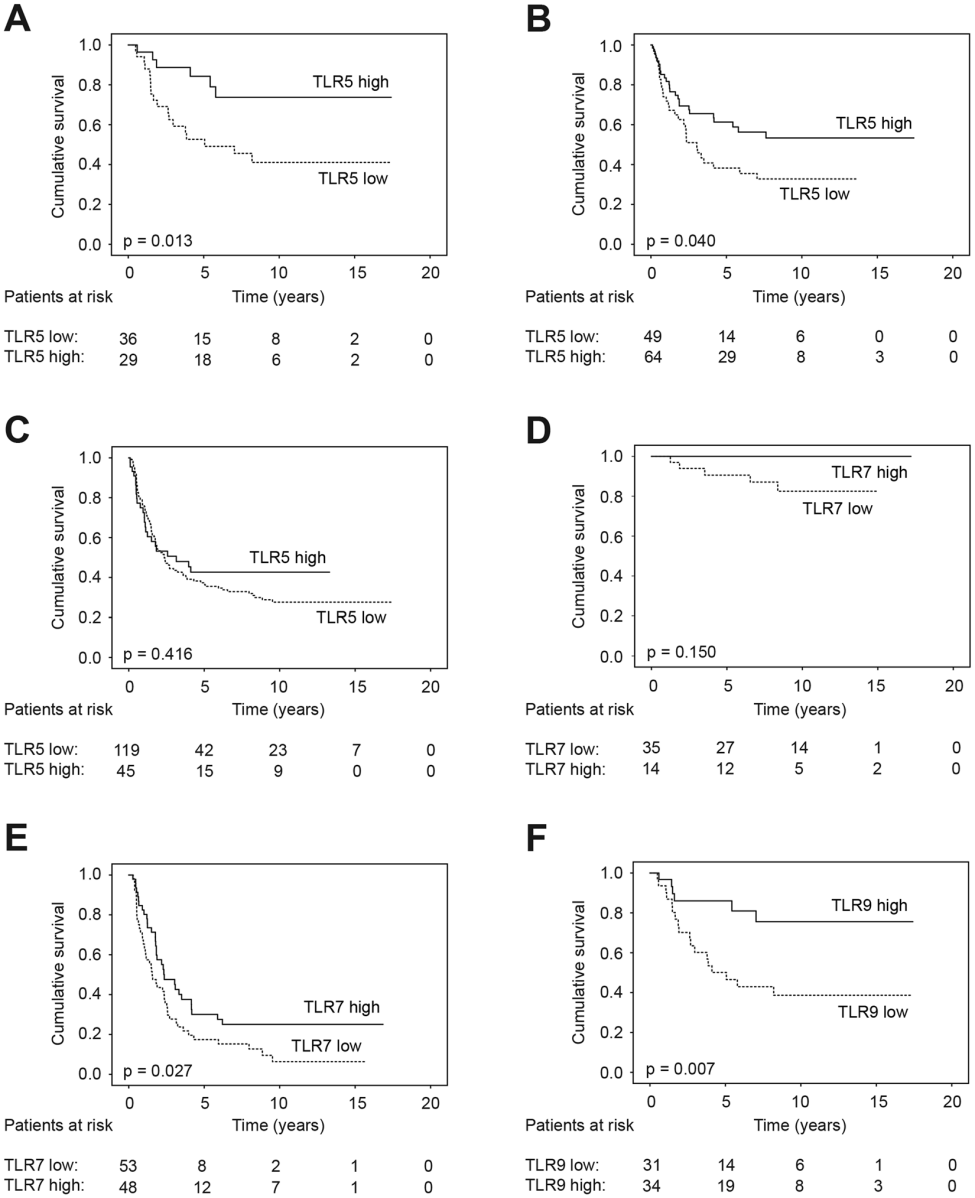
Gastric cancer patients’ disease-specific survival by subgroup according to the Kaplan–Meier method, with p values for significance based on the log-rank test. A high versus a low TLR5 expression amongst patients with (**A**) stage II disease, (**B**) an intestinal-, and (**C**) a diffuse-type cancer. A high versus a low TLR7 expression amongst patients with (**D**) stage I and (**E**) stage III disease. (**F**) A high versus a low TLR9 expression amongst patients with stage II disease.

In the subgroup analyses, a high TLR5 expression emerged as an indicator of a better prognosis amongst patients with stage II disease (hazard ratio [HR] 0.33; 95% CI 0.13–0.83; p = 0.019; Fig. 3A), amongst younger patients (HR 0.55; 95% CI 0.32–0.95; p = 0.033), amongst those with no distant metastasis (HR 0.66; 95% CI 0.44–0.99; p = 0.044), and amongst those with an intestinal-type cancer (HR 0.58; 95% CI 0.34–0.98; p = 0.043: Fig. 3B), but not amongst those with a diffuse-type cancer (HR 0.83; 95% CI 0.53–1.31; p = 0.417; Table 6 and Fig. 3C). Amongst patients with a high TLR7 expression and stage I disease, no deaths due to gastric cancer were recorded during our follow-up period (HR 0.03; 95% CI 0.01–97.7; p = 0.392; Fig. 3D). Moreover, a high TLR7 expression indicated a better prognosis amongst patients with stage III disease (HR 0.60; 95% CI 0.38–0.95; p = 0.029; Fig. 3E), amongst those with a pT4 tumour (HR 0.51; 95% CI 0.32–0.80, p = 0.003), and amongst those with lymph-node metastasis (HR 0.67; 95% CI 0.47–0.96; p = 0.029). Furthermore, a high TLR9 expression indicated a better prognosis amongst patients with stage II disease (HR 0.30; 95% CI 0.12–0.76; p = 0.011; Fig. 3F). Lastly, TLR1, TLR2, and TLR4 expression levels did not significantly predict the outcome in any patient subgroup (Supplementary Table 2).

**Table 6:** Survival analyses by subgroups, high TLR5, TLR7, and TLR9 expressions compared to low in 313 gastric cancer patients.

	High TLR5			High TLR7			High TLR9		
	HR	95% CI	p value	HR	95% CI	p value	HR	95% CI	p value
Age, years									
<67	0.55	0.32–0.95	0.033	1.07	0.67–1.73	0.767	0.90	0.56–1.43	0.648
≥67	0.66	0.42–1.02	0.059	0.72	0.47–1.10	0.131	0.69	0.45–1.05	0.086
Gender									
Male	0.68	0.42–1.10	0.114	0.98	0.62–1.56	0.932	0.82	0.52–1.30	0.400
Female	0.63	0.39–1.02	0.058	0.84	0.54–1.31	0.443	0.87	0.57–1.34	0.534
Stage									
I	0.41	0.05–3.67	0.423	0.03	0.01–97.7	0.392	1.59	0.26–9.63	0.613
II	0.33	0.13–0.83	0.019	0.79	0.35–1.79	0.572	0.30	0.12–0.76	0.011
III	0.96	0.60–1.52	0.853	0.60	0.38–0.95	0.029	0.93	0.60–1.44	0.728
IV	0.97	0.51–1.85	0.923	0.87	0.48–1.56	0.634	1.23	0.71–2.16	0.461
Tumour classification (pT)									
pT1	0.44	0.05–4.23	0.476	N/A			0.34	0.04–3.25	0.348
pT2	0.72	0.19–2.73	0.629	0.99	0.30–3.24	0.984	1.55	0.46–5.21	0.476
pT3	0.58	0.33–1.01	0.056	0.93	0.55–1.57	0.798	0.99	0.59–1.66	0.956
pT4	0.76	0.48–1.19	0.229	0.51	0.32–0.80	0.003	0.86	0.56–1.31	0.470
Lymph node metastasis (pN)									
pN0	0.69	0.31–1.54	0.364	1.66	0.78–3.53	0.190	0.67	0.31–1.47	0.317
pN1–3	0.69	0.47–1.02	0.062	0.67	0.47–0.96	0.029	0.81	0.57–1.16	0.250
Distant metastasis (pM)									
pM0	0.66	0.44–0.99	0.044	0.82	0.56–1.20	0.307	0.73	0.50–1.06	0.101
pM1	0.97	0.51–1.85	0.923	0.87	0.48–1.56	0.634	1.23	0.71–2.16	0.461
Laurén classification									
Intestinal	0.58	0.34–0.98	0.043	1.06	0.58–1.96	0.844	0.80	0.44–1.45	0.460
Diffuse	0.83	0.53–1.31	0.417	1.05	0.69–1.62	0.812	1.06	0.70–1.58	0.794

Abbreviations: TLR = Toll-like receptor, CI = Confidence interval, HR = Hazard ratio, N/A = not available.

## Discussion

In this study, we show for the first time that a high TLR5 tissue expression may identify gastric cancer patients with a favourable outcome, particularly amongst those with stage II disease, an intestinal-type cancer, without distant metastases or a younger age. In addition to TLR5, TLR1, TLR2, TLR4, TLR7, and TLR9 were also expressed in gastric cancer tissues, yet their expression levels did not function as prognostic biomarkers across the entire patient cohort. Furthermore, we showed that a high TLR7 expression may identify patients with a better prognosis amongst those with an advanced disease. High expressions of each of TLR studied associated with an intestinal-type cancer, suggesting that the inflammatory activity in the gastric mucosa is stronger in intestinal- than in diffuse-type tumours. Moreover, the high expressions of each TLR studied also associated with high expressions of all other TLRs.

In comparison to our results, Park *et al*.^14^ found that TLR5 activation via flagellin enhanced the proliferation of gastric cancer cells *in vitro*. Our results differ through the application of immunohistochemistry on surgical patient samples, where we show that the prognosis amongst gastric cancer patients with a high TLR5 expression is better than amongst those with a low tumour tissue expression. This discrepancy may result from the fact that Park *et al*. completed *in vitro* studies, whereas we conducted an *in vivo* study, rending the results not directly comparable.

TLR3, TLR4, and TLR9 were previously studied in a smaller gastric cancer patient series using a methodology similar to ours, relying on immunohistochemistry and tissue microarrays^12^. Similar to our findings, that study concluded that TLR4 and TLR9 expression levels did not significantly predict outcome in gastric cancer patients. Interestingly, in that study, a high TLR3 expression appeared to associate with a poor prognosis. Unfortunately, TLR3 was not included in our panel of biomarkers. In another study amongst 47 gastric cancer samples studied using immunohistochemistry, a high TLR2 expression associated with metastatic disease^13^. We found that a high TLR2 expression associated with an intestinal-type cancer, but not with any other clinicopathological variables. In the previous study, quantification of TLR expression levels relied on both the intensity and percentage of stained cells, whereas we only evaluated staining intensity.

TLR7 was previously thought to reduce the viability of gastric cancer cells^15^. Accordingly, we found that prognosis was better amongst patients with a high TLR7 expression in several subgroups, particularly among those with locally advanced disease. Interestingly, we recorded no gastric cancer–related deaths among stage I patients with a high TLR7 expression. Although patients with stage I disease typically enjoy a good overall prognosis, it is very surprising that no deaths due to gastric cancer were recorded. Unfortunately, the number of patients in this subgroup was too small to draw definitive conclusions; our results, however, encourage further study of TLR7 expression in early-stage gastric cancer.

TLR-related therapy, particularly TLR agonists, capable of activating the immune system against cancer have been broadly studied in several malignancies^18,19^. The administration of a TLR7 agonist, imiquimod, increases the expression of TLR7 in gastric cancer cells and reduces their viability^15^. Whether imiquimod has direct cytotoxic effects or if acting via the modulation of inflammatory cell activity remain incompletely understood since numerous different pathways are involved. *In vitro* studies of imiquimod on colon and basal cell carcinoma cells suggest that it can directly induce cell death^20,21^.

In addition, gastric cancer predisposing *H*. *pylori* infection induces polymorphous TLR expression in the gastric mucosa, since TLRs are essential for immunity against it^22–25^. Genetic variations of TLR1, TLR5, and TLR9 may contribute to the malignant transformation of the gastric mucosa by altering the immune response to *H*. *pylori*^16,26,27^. On the other hand, a TLR2 polymorphism was recently shown to function as a potential prognostic biomarker in gastric cancer patients independent *of H*. *pylori* infection status, suggesting that gastric carcinogenesis affecting signalling pathways does not merely limit to crosstalk with *H*. *pylori*^28^. Conversely, we identified no TLR2 protein expression that functioned as a prognostic biomarker in gastric cancer patients, although the results related to protein expression and gene polymorphism analyses are not directly comparable. Unfortunately, in our retrospective study, patients’ serum antibody levels against *H*. *pylori* were unavailable and the *H*. *pylori* infection status from the archived tissue samples was impossible to reliably determine.

The strengths of this study include the large patient cohort with precise and reliable follow-up information and the uniformity of laboratory methods used to determine the expression levels of TLRs. Yet, the single-centre setting introduces a bias and limits the generalisability of our results. Additional studies on other well-defined patient cohorts are needed in order to validate our results. Furthermore, in addition to *H*. *pylori* infection status, the limitations of this study include the lack of information for certain other well-known risk factors in our gastric cancer cases, such as venous and perineural invasion, lymphatic emboli, and the tumour subsite. Accessing details regarding each of these factors in a retrospective manner may potentially introduce inaccuracies and, thus, we did not include them in our analyses. Automated digital scoring systems have proved beneficial in improving the reproducibility of evaluating the visual density of immunohistochemically stained samples^29^. Some automated digital systems can already identify individual cells; however, the available systems cannot yet reliably interpret the cells’ morphological features, and, thus, identify malignant cells from the stroma^30–32^. Studying heterogeneous tissues, reliably identifying neoplastic cells from non-neoplastic cells remain essential in order to produce reproducible data. Furthermore, in the context of toll-like receptors, the tumour microenvironment’s immune cell toll-like receptor expression limits the application of the digital colourimetric quantification of tissue cores. Thus, in this study, digital colourimetric quantification of tissue cores was, unfortunately, not possible.

To conclude, in this study we show, for the first time, that a high TLR5 tissue expression may identify gastric cancer patients with a better prognosis, particularly amongst those with a stage II disease or an intestinal-type cancer. In a small subgroup of stage I disease, none of the patients with a high TLR7 expression died from gastric cancer. In addition, we found that a high expression of TLR1, TLR2, TLR4, TLR5, TLR7, and TLR9 associated with an intestinal-type gastric cancer and with a high expression of all other TLRs.

## Supplementary information

Supplemetary Tables 1 and 2

## Data Availability

All data and materials are available from the corresponding author upon request.
