# Supplementary material for: Evaluation of toll-like receptors as prognostic biomarkers in gastric cancer: high tissue TLR5 predicts a better outcome
Source: Sci Rep. 2019 Aug 29;9:12553. doi: 10.1038/s41598-019-49111-2 (PMC6715705; doi:10.1038/s41598-019-49111-2)
Supplement: Supplementary file 1 — Supplemetary Tables 1 and 2 [file 41598_2019_49111_MOESM1_ESM.pdf]

Evaluation of toll-like receptors as prognostic biomarkers in gastric cancer: high tissue TLR5 predicts a better outcome

Aaro Kasurinen[1*], Jaana Hagström[2], Alli Laitinen[1,3], Arto Kokkola[3], Camilla Böckelman[1,3**] & Caj Haglund[1,3**]

[1]Translational Cancer Medicine Research Programme, University of Helsinki, Helsinki, Finland

[2]Department of Pathology and Oral Pathology, University of Helsinki and Helsinki University Hospital, Helsinki, Finland

[3]Department of Surgery, University of Helsinki and Helsinki University Hospital, Helsinki, Finland

*Correspondence and requests for materials should be addressed to Aa.K. (email: aaro.kasurinen@helsinki.fi)

**Authors contributed equally to this work.

Supplementary Table 1. Association of TLR1, TLR2, TLR4, TLR5, TLR7, and TLR9 expressions among each other in 313 gastric cancer patients.

	**TLR1**			**TLR2**			**TLR4**			**TLR5**			**TLR7**		
	**Low (%)**	**High (%)**	**p value[1]**	**Low (%)**	**High (%)**	**p value[1]**	**Low (%)**	**High (%)**	**p value[1]**	**Low (%)**	**High (%)**	**p value[1]**	**Low (%)**	**High (%)**	**p value[1]**
TLR2															
Low	73 (67.0)	36 (33.0)	<0.001												
High	58 (34.9)	108 (65.1)													
TLR4															
Low	69 (68.3)	32 (31.7)	<0.001	66 (68.8)	30 (31.3)	<0.001									
High	68 (37.8)	112 (62.2)		43 (24.0)	136 (76.0)										
TLR5															
Low	104 (61.9)	64 (38.1)	<0.001	82 (50.3)	81 (49.7)	<0.001	73 (43.7)	94 (56.3)	<0.001						
High	29 (26.6)	80 (73.4)		25 (23.1)	83 (76.9)		25 (22.9)	84 (77.1)							
TLR7															
Low	87 (60.8)	56 (39.2)	<0.001	79 (56.0)	62 (44.0)	<0.001	69 (48.3)	74 (51.7)	<0.001	100 (70.9)	41 (29.1)	<0.001			
High	39 (31.2)	86 (68.8)		21 (17.1)	102 (82.9)		21 (16.8)	104 (83.2)		57 (46.0)	67 (54.0)				
TLR9															
Low	94 (70.1)	40 (29.9)	<0.001	77 (59.2)	53 (40.8)	<0.001	70 (52.6)	63 (47.4)	<0.001	97 (74.0)	34 (26.0)	<0.001	88 (71.5)	35 (28.5)	<0.001
High	40 (28.0)	103 (72.0)		31 (21.7)	112 (78.3)		26 (18.2)	117 (81.8)		68 (47.9)	74 (52.1)		54 (37.8)	89 (62.2)	

Abbreviations: TLR = Toll-like receptor

[1]Pearson's Chi–squared test

Supplementary Table 2. Survival analyses by subgroups, high TLR1, TLR2, and TLR4 expressions compared to low in 313 gastric cancer patients.

	High TLR1			High TLR2			High TLR4		
	**HR**	**95% CI**	**p value**	**HR**	**95% CI**	**p value**	**HR**	**95% CI**	**p value**
Age, years									
<67	0.77	0.49–1.22	0.272	0.73	0.47–1.15	0.176	0.64	0.41–1.01	0.053
≥67	0.82	0.54–1.26	0.367	1.17	0.74–1.85	0.497	0.97	0.62–1.51	0.891
Gender									
Male	0.89	0.56–1.41	0.620	1.04	0.65–1.68	0.872	0.72	0.46–1.15	0.167
Female	0.78	0.51–1.18	0.235	0.89	0.58–1.35	0.572	0.88	0.57–1.35	0.546
Stage									
I	3.22	0.36–29.0	0.298	3.80	0.42–34.1	0.233	0.53	0.09–3.16	0.482
II	0.46	0.20–1.08	0.076	0.56	0.25–1.24	0.151	0.69	0.29–1.61	0.388
III	1.02	0.66–1.59	0.992	0.70	0.45–1.09	0.115	0.72	0.46–1.13	0.154
IV	1.35	0.78–2.33	0.286	0.95	0.52–1.74	0.867	1.23	0.71–2.14	0.454
Tumour classification (pT)									
pT1	0.94	0.13–6.71	0.953	1.41	0.20–9.99	0.733	0.32	0.03–3.06	0.321
pT2	0.63	0.20–1.98	0.427	0.35	0.11–1.09	0.070	0.32	0.10–1.02	0.053
pT3	0.68	0.41–1.14	0.141	1.02	0.60–1.73	0.954	1.01	0.57–1.80	0.966
pT4	1.27	0.83–1.93	0.277	0.79	0.51–1.22	0.285	0.69	0.45–1.05	0.086
Lymph node metastasis (pN)									
pN0	0.76	0.36–1.61	0.471	1.29	0.60–2.78	0.518	1.20	0.54–2.68	0.649
pN1–3	1.00	0.70–1.42	0.991	0.74	0.52–1.06	0.097	0.74	0.52–1.05	0.093
Distant metastasis (pM)									
pM0	0.70	0.48–1.02	0.066	0.86	0.59–1.25	0.428	0.81	0.55–1.18	0.267
pM1	1.35	0.78–2.33	0.286	0.95	0.52–1.74	0.867	1.23	0.71–2.14	0.454
Laurén classification									
Intestinal	0.73	0.43–1.26	0.264	0.92	0.46–1.82	0.802	1.08	0.54–2.14	0.835
Diffuse	1.00	0.68–1.47	0.984	1.19	0.81–1.74	0.377	0.82	0.56–1.20	0.301

Abbreviations: TLR = Toll-like receptor, CI = Confidence interval, HR = Hazard ratio
